# Impact of radiofrequency ablation (RFA) on bone quality in a murine model of bone metastases

**DOI:** 10.1371/journal.pone.0256076

**Published:** 2021-09-08

**Authors:** Soroush Ghomashchi, Cari M. Whyne, Tricia Chinnery, Fayez Habach, Margarete K. Akens

**Affiliations:** 1 Department of Medical Biophysics, University of Toronto, Toronto, Ontario, Canada; 2 Sunnybrook Research Institute, Toronto, Ontario, Canada; 3 Department of Surgery, University of Toronto, Toronto, Ontario, Canada; 4 Techna Institute, University Health Network, Toronto, Ontario, Canada; 5 Department of Physics, University of Toronto, Ontario, Canada; University of Life Sciences in Lublin, POLAND

## Abstract

Thermal therapies such as radiofrequency ablation (RFA) are gaining widespread clinical adoption in the local treatment of skeletal metastases. RFA has been shown to successfully destroy tumor cells, yet the impact of RFA on the quality of the surrounding bone has not been well characterized. RFA treatment was performed on femora of rats with bone metastases (osteolytic and osteoblastic) and healthy age matched rats. Histopathology, second harmonic generation imaging and backscatter electron imaging were used to characterize changes in the structure, organic and mineral components of the bone after RFA. RFA treatment was shown to be effective in targeting tumor cells and promoting subsequent new bone formation without impacting the surrounding bone negatively. Mineralization profiles of metastatic models were significantly improved post-RFA treatment with respect to mineral content and homogeneity, suggesting a positive impact of RFA treatment on the quality of cancer involved bone. Evaluating the impact of RFA on bone quality is important in directing the growth of this minimally invasive therapeutic approach with respect to fracture risk assessment, patient selection, and multimodal treatment planning.

## Introduction

Skeletal bone metastases occur in one third of all cancer [1] with frequent complications affecting 85 percent of patients [2]. Radiofrequency ablation (RFA) is a localized, minimally invasive therapeutic treatment that is used clinically to ablate cancerous tissue, such as osteoid osteoma [2–4] and soft tissue lesions. More recently, cancer patients with skeletal metastases exhibiting pain have been treated with RFA [5–7]. The goal of this treatment is pain palliation and localized tumor growth control. It is often used in conjunction with cement augmentation procedures [8]. However, the impact of RFA itself on bone quality is unknown.

In its use, a radiofrequency (RF) generator supplies an alternating current to a probe inserted into the tissue to be treated. Frictional heat induced by the electric current creates a thermal ablation zone. The RF circuit may be completed using grounding pads in unipolar designs [9] or with grounding included on bipolar probes [10]. Efficacy and safety of RFA has been established and multiple probe designs have overcome earlier shortcomings of this technology such as charring and boiling injuries, incomplete RF circuitry, and have enabled the ablation of larger lesions [10–12].

RFA leads to a coagulative necrosis within seconds to minutes and distinct defined tissue changes are visible after RFA dose delivery [13]. Typically, this is seen with signs of micro-cavitation or charring along the needle track, necrotic regions highlighted by a dark hemorrhage ring, and an RFA-treated lesion boundary with signs of inflammation, edema, and hyperemia [9, 13]. In addition to RF probe design and applied parameters such as diameter probe, lengths, and temperature the degree of the treatment effect is influenced by tissue properties such as vascularity and electric conductivity. Conductivity is low in bone protecting the surrounding tissue when RF is applied within the bone.

Architecture and tissue level material properties of bone impact its strength and are regulated through the mineral (hydroxyapatite, HA) and organic (primarily collagen) components of their ultrastructure [14–16]. The inner and intrafibrillar spacing within the rectilinear structure of collagen type I fibrils is filled with HA crystals [17]. HA is a thermodynamic ally stable, hexagonal structure of calcium phosphate [18]. Mineral content of HA crystals, along with their size, is modified over time due to the time-dependent nature of HA crystallization [19]. The mineral phase of bone controls its strength and stiffness [20]. Bone mineral density distribution (BMDD) is an indicator of bone quality [21, 22].

In the organic component of bone tissue, the distribution and orientation of collagen type I fibrils are responsible for bone’s ductility and toughness [17]. The rectilinear fibrillar collagen structure is formed by covalent enzymatic/non-enzymatic cross-links [23, 24]. Alterations in the efficacy and presence of cross-links is associated with modifications in bone’s ductility and toughness [25–27]. Collagen structure has been shown to be influenced by presence of different types of bone metastases [28] with respect to fibril organization and morphology.

Previous work by our group demonstrated different effects of various types of metastases on rat bone quality [27, 29–31]. Limited work has been done to examine the impact of RFA on healthy bone [32], but the impact of RFA on bone with metastases has not previously been investigated. This study aims to quantify the impact of RFA on bone quality in both healthy and metastatically involved bone. It is hypothesized that RFA does not negatively impact the quality of healthy and metastatic bone. An understanding of the impact of RFA treatment on bone quality is important in the context of fracture risk assessment and its incorporation in clinical multimodal treatment planning for bone metastases.

## Material and methods

### Animal model and metastatic inoculation

**Fig 1:**
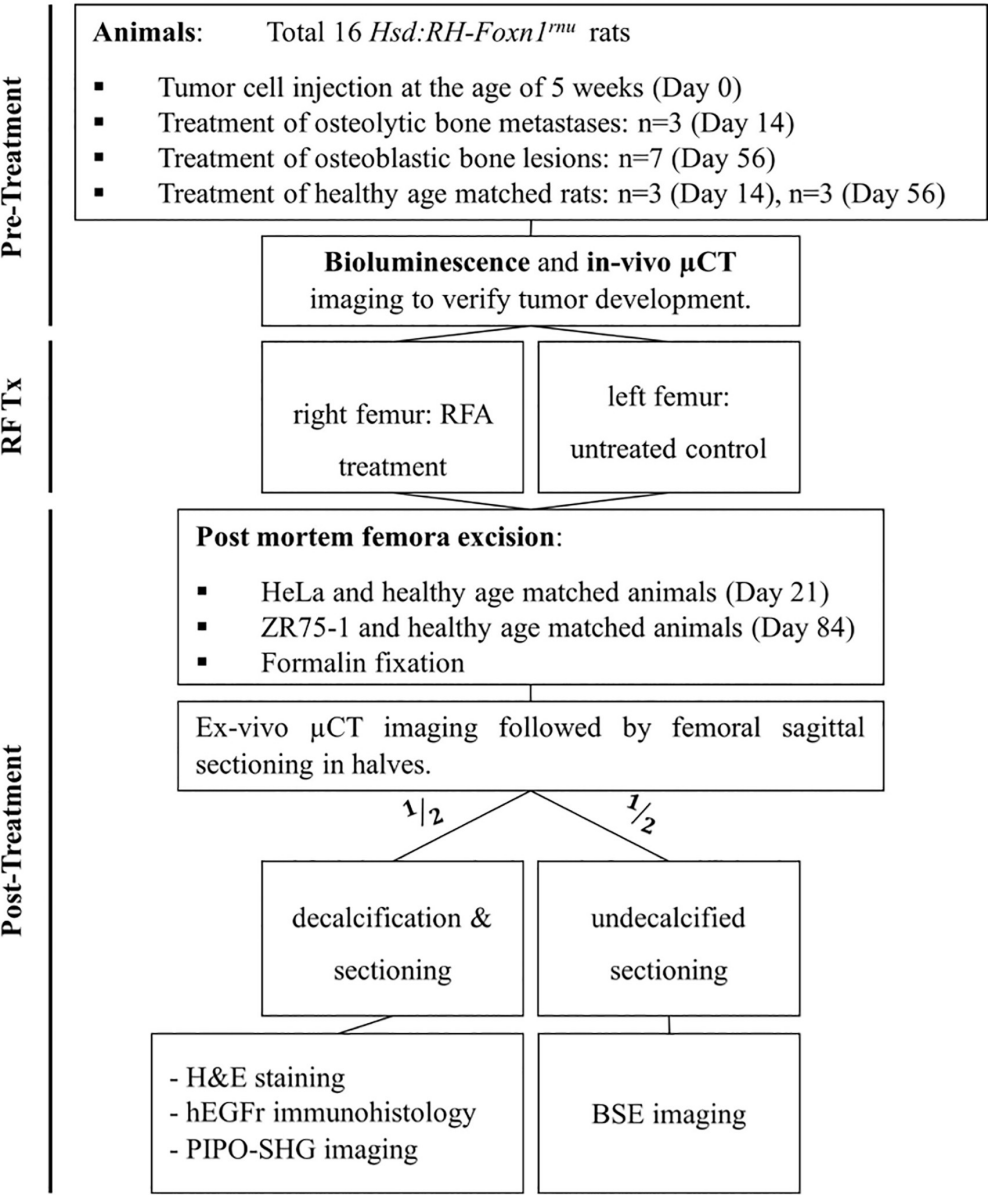
Experimental design and workflow. Micro computer tomography (μCT); radiofrequency ablation treatment (RF Tx); radiofrequency ablation (RFA); hematoxylin and eosin (H&E); human epithelium growth factor receptor (hEGFr); polarization-in, polarization-out (PIPO) second harmonic generation (SHG); backscatter electron (BSE).

With approval from the University Health Network animal care committee (AUP 6044), human tumor cells were injected into 5-week old athymic female Hsd:RH-*Foxn1*^*rnu*^ rats (Envigo, Indianapolis, IN, USA) through intracardiac injection under general inhalation anaesthesia. The intracardiac injection of luciferase transfected human ZR-75-1 (ATCC® CRL-1500™) breast cancer cells resulted in the development of osteoblastic metastases within 56 days. Intra-cardiac injection of 1.5 x 10^6^ luciferase transfected HeLa cells [33, 34] produced osteolytic bone metastases within 14 days in the athymic rats. All the rats injected in this study developed metastases at the presented timelines (Fig 1). The different age of the rats at onset of bone metastases (osteoblastic vs osteolytic) required the use of age-matched healthy animals due to age-dependent differences in bone remodeling.

### Bioluminescent Imaging (BLI) and μCT scanning

The luciferase transfected cancer cell lines allowed for monitoring tumor development and progression via bioluminescent imaging (IVIS Spectrum, Perkin Elmer). BLI was performed every 21- or 7-days post inoculation in the osteoblastic and osteolytic groups, respectively. Prior to imaging, luciferin (XenoLight D-Luciferin, PerkinElmer, Canada) was prepared at a concentration of 40 mg/ml 0.9% sodium chloride and intra-peritoneally injected in each rat (90 mg/kg). Peak intensity of the signals is achieved after 13 minutes. Prior to treatment delivery, in-vivo μCT scanning (Locus Ultra μCT, GE, 154μm resolution) was utilized to locate metastatic lesions.

### Radiofrequency ablation (RFA)

Post inoculation, due to the differing timelines of bone metastases development, rats with osteoblastic and osteolytic bone lesions were treated with RFA on days 14 and 56, respectively. Age-matched healthy rats were treated according to the same timeline (Fig 1). In inoculated rats, metastatic lesions were formed in both femora. As such, in each rat (metastatically involved and age-matched healthy animals), the right femur was treated with RFA and the contralateral femur was left untreated and served as intra-animal control. The femur was chosen due to its size and intramedullary access. After the RFA treatments the rats received analgesics 0.1 mg/kg meloxicam subcutaneously (s.c.) (Metacam, Boehringer Ingelheim, CT, US) and were monitored for clinical symptoms (level of activity, skin changes, weight changes, and general appearance). All treatments and imaging were performed under general anaesthesia with isoflurane and oxygen (1.5–2.5%; 2L).

The RFA system utilized in this study was comprised of a custom designed 22 G (0.64 mm outer diameter) monopolar probe integrated with an RFA generator (Baylis Medical Company, Mississauga, ON, Canada). Heat shrink was applied onto the needle for impedance control. The tip of the introducer (i.e. where the active portion of the probe sticks out) was left exposed to allow for completion of a full circuit. The monopolar probe incorporated a thermocouple at the active tip to allow for real-time monitoring of fluctuations in supplied temperature. Current was delivered from the generator at a maximum power output of 3 Watts. A controlled ramp rate of 40 ˚C/min was used with a target temperature of 65 ˚C. Length of treatment was controlled by the generator and set to 3 minutes. These parameters were established in a pilot study to ensure lesion size able to span the width of the femur but did not impair the surrounding soft tissue.

Under general anaesthesia, each rat was placed in dorsal recumbency with the right knee bent and the skin around the knee joint prepared for surgery. A 1 cm lateral para patellar skin incision was performed prior to inserting an 18 G introducer between the femoral condyles into the femoral canal, followed by the RF-probe insertion. The position of the probe within the canal was confirmed using fluoroscopic guidance (OEC 9800 Plus Mobile C-arm, GE Healthcare, Mississauga, ON, Canada). Ablated temperature and heat dissipation to the surrounding tissue was monitored via a thermographic camera (FLIR C2, FLIR Systems Inc, Burlington, ON, Canada) or an infrared thermometer non-contact laser temperature gun (surface measurements; Tacklife, Toronto, ON, Canada).

After the treatment, the probe was removed, the skin incision was stapled using the AutoClip system (AutoClip® System, Fine Science Tools, B.C., Canada) and a subcutaneous injection of 0.1 mg/kg meloxicam (Metacam, Boehringer Ingelheim, CT, US) was given to each animal. In some cases where animals experienced prolonged pain, 0.05 mg/kg of buprenorphine (Temgesic, Schering-Plough, NJ, US) was administered. An antibacterial ointment (Flamazine; Smith and Nephew, Bowers Medical, Burlington, ON, Canada) was applied to the skin around the stapled area.

Rats were euthanized using CO_2_ asphyxiation at day 21 and 84 post-RF treatment depending on the type of treated bone lesions (Fig 1). The appendicular bones (femora and tibiae) were excised, either wrapped in saline-soaked sponges and stored at -20˚C or fixed in 10% buffered formalin. At the timepoint of dissection no macroscopic changes to the tissue surrounding the femur have been observed. At the endpoint of each cohort, the excised femora underwent μCT imaging (μCT 100, Scanco Medical, Southeastern, PA, USA) at a voxel size of 20 μm.

### Specimen preparation and histopathologic analysis

In each rat, formalin fixed femora were cut in half along the sagittal plane. One half of each specimen was decalcified in 10% ethylenediaminetetraacetic acid (EDTA) solution and the other corresponding non-decalcified halves were embedded in Spurr’s epoxy resin.

Decalcified specimens were dehydrated, embedded in paraffin and 5 μm sections were stained with Hematoxylin and Eosin (H&E) for standard histology analyses focusing on changes to the bone, tumor cell distribution and for SHG analyses [35]. In the osteolytic cohort, the human EGFr receptor present on the HeLa cells was stained using immunohistochemistry to identify the tumor more easily within the bone tissue [33].

### Polarimetric Second Harmonic Generation (SHG) imaging

Polarization-dependent Second Harmonic Generation (P-SHG) is being used to analyze the collagen structure through examining the orientation of fiber axis and second-order susceptibility ratio [28]. Second-order susceptibility ratio emphasizes the significance of alterations in spatial symmetries and distribution of collagen fibers. Regions of interest (ROI) were selected on the H&E slides for linear polarization-in, polarization-out (PIPO) SHG microscopy imaging. ROIs were chosen in multiple locations throughout the trabecular centrum which were marked as RFA-treated in the presence and absence of metastasis. The linear PIPO SHG microscopy was performed using an in-house developed laser scanning non-linear microscope (Barzda Biophysics, UTM, Mississauga, ON, Canada). Characterization of collagen fibril orientation and distribution in non-pathologic and metastatic bone tissue has been established using SHG imaging (i.e. susceptibility ratio) [27, 29].

### Backscattered electron microscopy (BSE)

**Fig 2:**
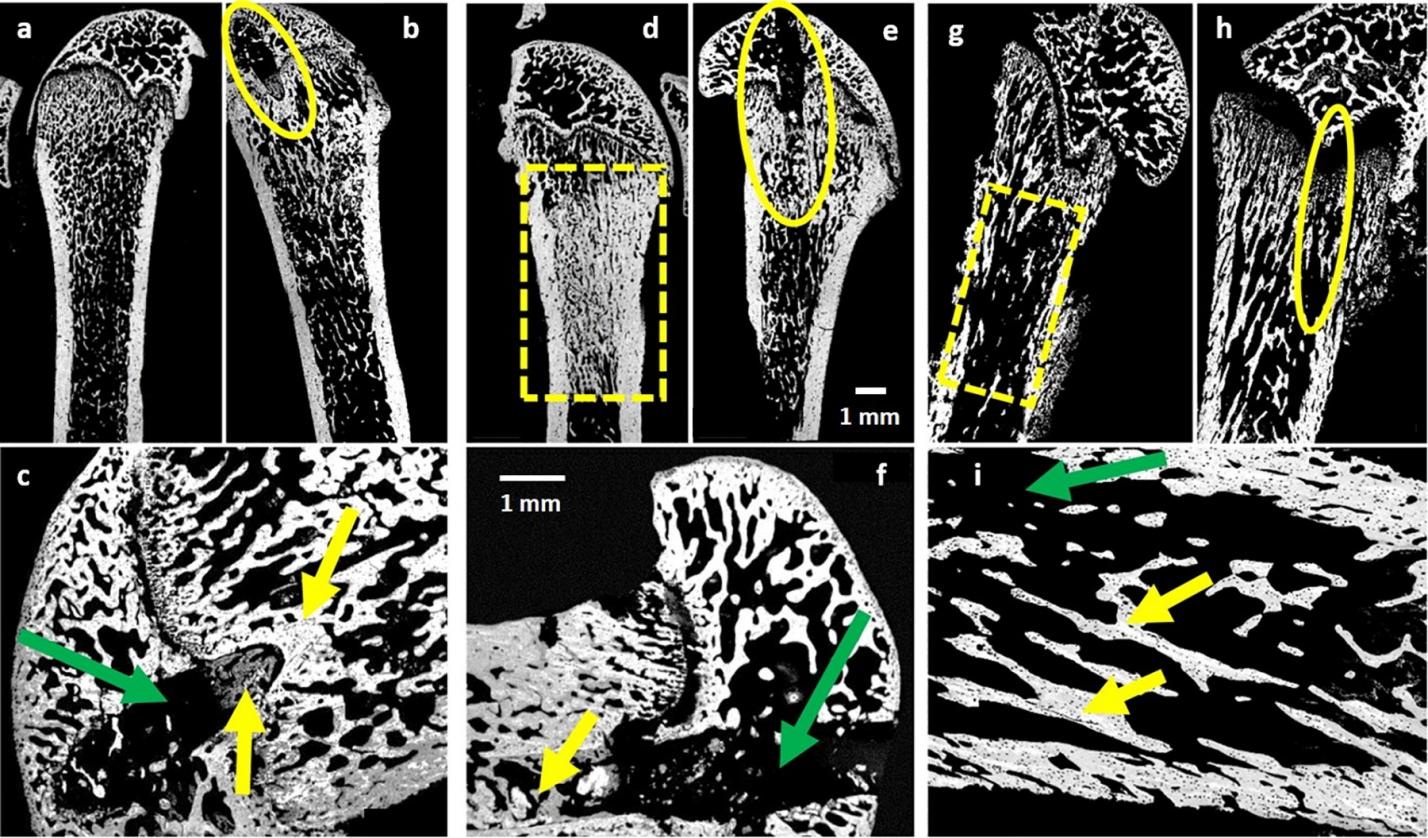
Backscatter electron images of rat femur. Representative sagittal cross-sectional BSE acquisitions. Regions of osteoblast proliferation, indicative of new bone formation, is seen post RFA (yellow arrows). Green arrows indicate probe insertion channels. a-c) Femora from healthy controls. d-f) Femora from ZR-75-1 cell injected rats: Osteoblastic-induced new bone formation is visualized identified inside the dashed rectangle box. g-i) Femora from HeLa cell injected rats: Osteolytic lesions in trabecular bone tissue demonstrate tumor involvement (i.e. dashed rectangular overlay).

Distribution of calcium contractions in bone tissue, indicative of mineral content, can be determined via BMDD measurements in quantitative BSE (qBSE) imaging. The second half of each femur (preserved in 10% buffered formalin solution) was immersed in varying acetone concentrations (70%, 90%, 100%) for sequential dehydration and then embedded in Spurr’s epoxy resin. Once cured, each block was microtomed to expose the bone surface, which was further polished using assorted paper grades (400, 600 and 1200 grit size) and diamond suspensions (6 μm and 1 μm). The polished surface of each block was carbon-coated and scanned via BSE microscopy (SS BSE detector, FEI, Hillsboro, Oregon, US) using an ESEM (FEI/Philips XL30, FEI, Hillsboro, Oregon, US). In each scan, BSE grey-levels were converted to calcium weight percentage [29, 31]. RFA-treated healthy and metastatic bone was identified in each specimen (Fig 2) and used to evaluate RFA-induced modifications in the mineralization profiles.

### Statistical analysis

For PIPO SHG and BSE data analyses, two sets of Welch’s t-tests were performed where the two independent groups were “Untreated” versus “Treated” in healthy and metastatic cohorts. In addition, a one-way ANOVA test was used to compare the four RFA-treated osteoblastic, osteolytic and corresponding age-matched cohorts. In all cases, data was presented as mean ± standard deviation (significance level of p<0.05). Conditions for assuming equal variance are reviewed in each case and the appropriate Welch’s t-test is then selected. Leven’s test was used to check for satisfaction of equal variances between means in each sample group.

## Results

### Clinical results

All rats tolerated the cell injection well and 100% of the injected rats developed either osteolytic (HeLa cells) or osteoblastic (ZR-75-1) bone lesions within both femora. The RFA treatment went well, and the animals did not show any unexpected clinical symptoms after the treatment. The age-matched healthy rats tolerated the RFA ablation well without showing unexpected clinical symptoms.

### Metastatic involvement and RFA treatment

Bioluminescent and ex-vivo μCT imaging verified tumor growth and progression respectively in the femora of all the inoculated rats (n = 10).

RFA was applied successfully within the femoral canal of all rats, metastatically involved (n = 10) and healthy (n = 6). After verification of the metastatic region via fluoroscopy, probe placement was consistent. Pre-treatment in-vivo μCT allowed for confirmation of the tumor location within the femur specific to each rat. Post euthanasia, no signs of excessive heat dissipation were found in the muscle surrounding the treated femora.

Thermocouple data captured from the generator demonstrated consistent delivery of RF dose with a targeted average maximum temperature of 65 ˚C. Ancillary thermocouple data from the camera or infrared thermocouple gun demonstrated consistent surface temperatures in the physiological range of 38.1 ˚C. The generator’s average output was recorded at 2.15 ± 0.13 W.

### Histopathologic analyses

**Fig 3:**
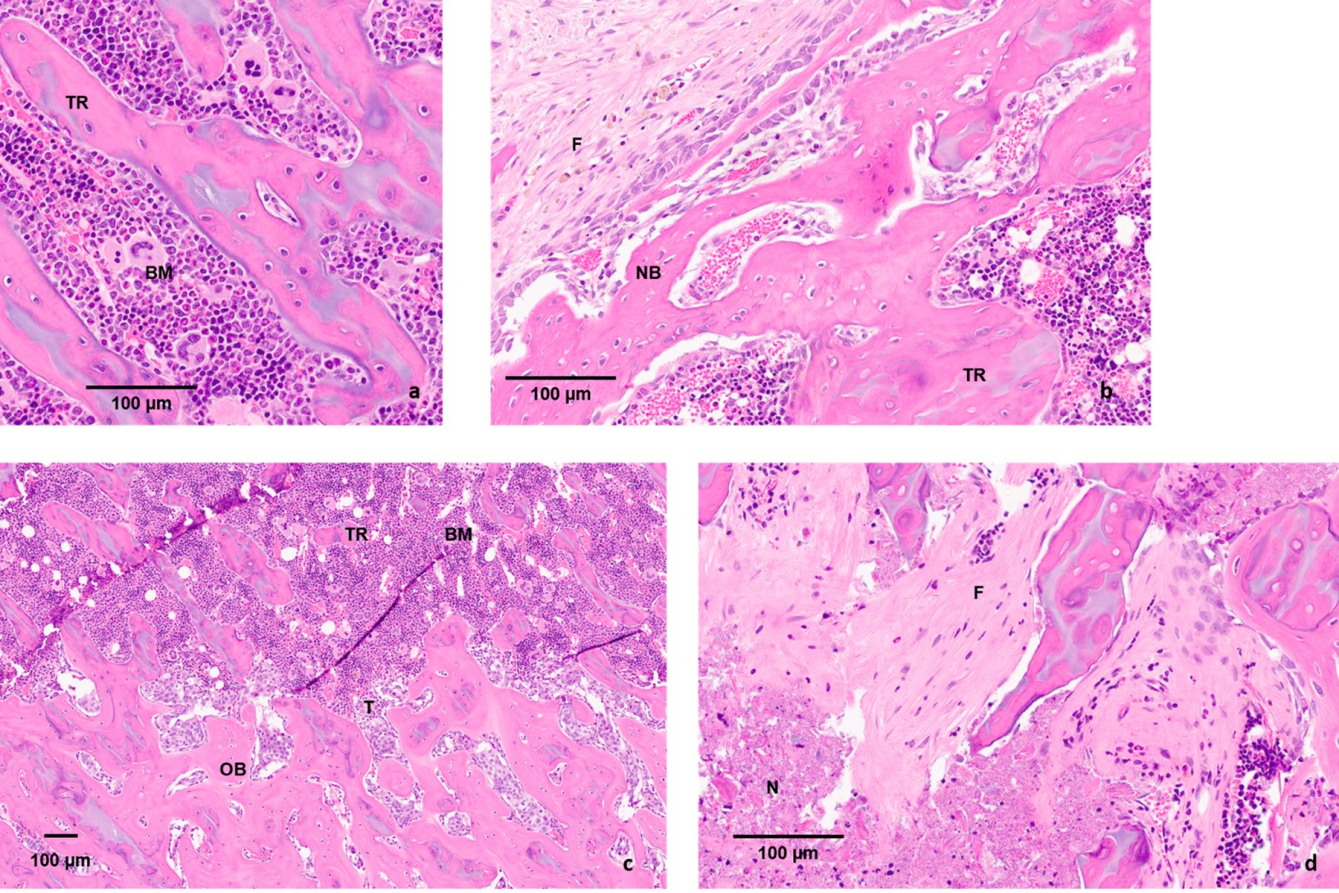
Histopathology of healthy bone tissue. Histology section of the femur of healthy control rats and rats with osteoblastic lesions: a) Mid-sagittal section of a distal femur in a healthy control rat stained with haematoxylin and eosin (BM: bone marrow; TR: trabeculae) b) Mid-sagittal section of a distal femur 4-week post RFA. Fibrous tissue (F) has formed within the treated region with the newly formed bone (NB) visible on the border of the treated area. c) Tumor cells (T; ZR-75-1) in the mid-sagittal section of a distal femur inducing osteoblastic bone formation (BM: bone marrow; TR: trabeculae; OB: osteoblastic bone formation) d) Mid-sagittal section of a distal femur 4 weeks post RFA. Tumor cells are necrotic (N) and fibrous tissue (F) has formed within the treated area.

**Fig 4:**
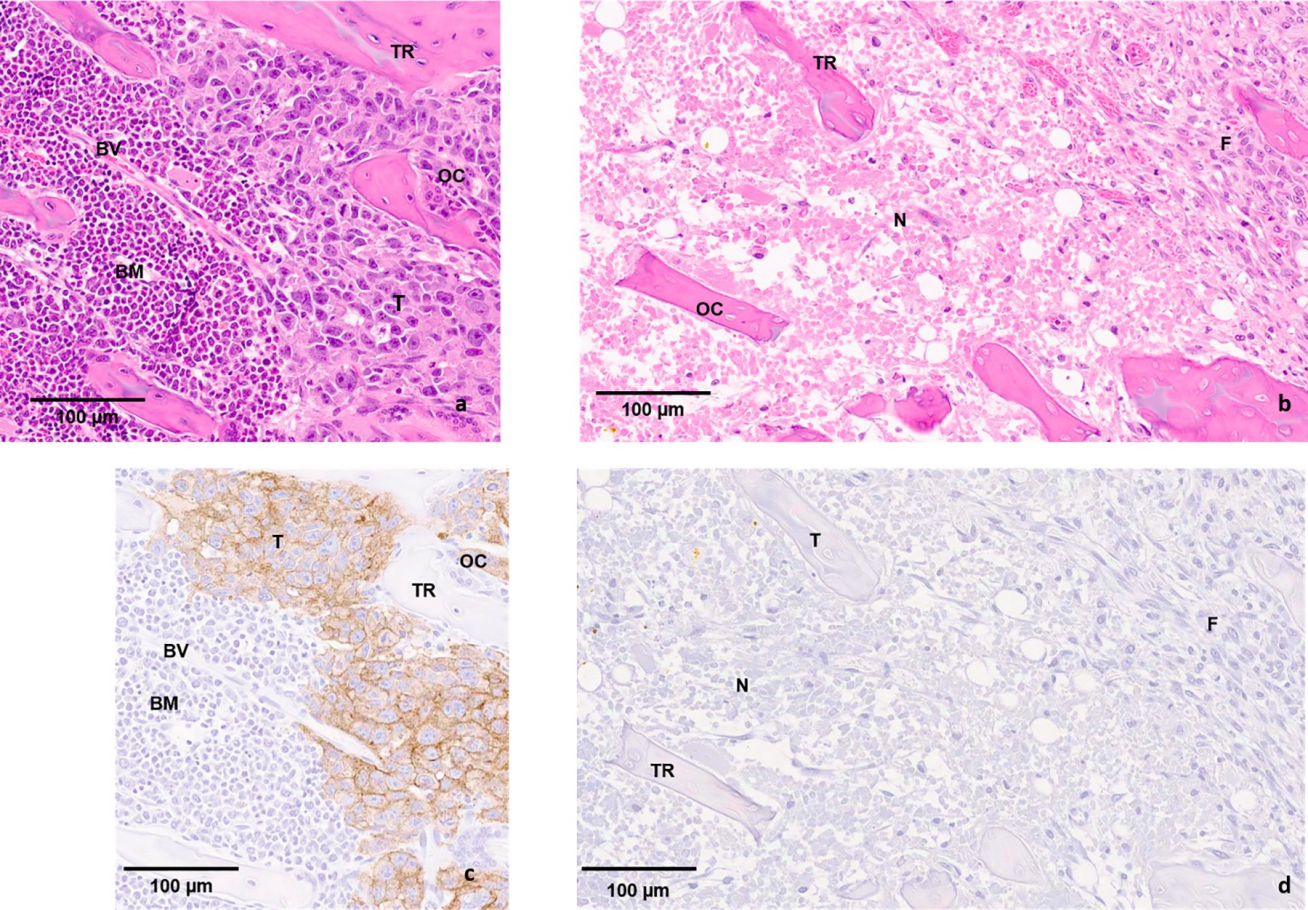
Histopathology of metastatic bone tissue. Histology section of the femur with osteolytic metastases: a) Tumor cells (T; HeLa) in the mid-sagittal section of a distal femur (BM: bone marrow; TR: trabeculae; OC: osteoclast; BV: blood vessel) b) Mid-sagittal section of a distal femur 1-week post RFA. Tumor cells are necrotic (N); osteocytes (OC) are dead within the trabeculae of the treated area surrounded by RFA-induced fibrous tissue (F) formation. c) Tumor cells (T) are staining positive for the hEGFr antibody in the mid-sagittal section of a distal femur (BM: bone marrow; TR: trabeculae; OC: osteoclast; BV: blood vessel) d) Mid-sagittal section of a distal femur 1 week post RFA. Tumor cells are necrotic (N), no longer stain positively and fibrous tissue (F) is formed within the treated area.

Histological analysis of the samples demonstrated distinct regions of RF effect in healthy and metastatic bone (Figs 3 and 4). At lower magnification, a clear demarcation associated with the RF ablated area was identified in comparison to the untreated regions. Examination at higher magnification revealed fibrous tissue formation replacing the bone marrow. Along the path of needle insertion and at the periphery of the ablated region, a mixture of live and dead osteocytes (empty lacunae) was present. Larger concentrations of dead cells (increased density of empty lacunae) were observed closer to the epicenter of the RFA-induced necrotic region. Along the distal and proximal ends of the RFA-treated area, more moderate necrosis was observed. Patches of RFA-treated lesions with live osteocytes were identifiable. Formation of fibrous tissue and inflammatory response was the most evidently observed RFA-induced effects inside the bone. The hEGFr stained histology slides from the osteolytic cohort showed RFA dose delivery resulted in an almost complete killing of the cancer cells (Fig 4).

### PIPO SHG microscopy

Orientation of collagen fibrils and its intrinsic properties determine the nonlinear susceptibility ratio [28]. Significantly higher susceptibility ratios were observed in untreated metastatic trabecular bone (osteoblastic and osteolytic, respectively) compared to untreated age-matched healthy bone tissue (Day 84: 2.473 vs. 2.420, p = 0.029; Day 21: 2.451 vs. 2.406, p = 0.027, Table 1). The susceptibility ratios were also significantly increased in RFA-treated age-matched healthy cohorts in comparison to the age-matched untreated healthy bone tissue (Day 84: 2.472 vs. 2.420, p = 0.003; Day 21: 2.472 vs. 2.406, p = 0.003, Table 1). Elevated susceptibility ratio values are associated with enhanced disorganization, disrupted in-plane alignment and increased degree of disorder in collagen type I fibrils. Statistically significant differences were not observed in RFA-treated metastatic cohorts compared to untreated metastatically involved bone tissue.

**Table 1:** Susceptibility Ratio (R).

Untreated	Mean ± Std.	RF Tx	Mean ± Std.
Healthy (Day 21)	2.403 ± 0.010	Healthy (Day 21)	2.472 ± 0.010
Osteolytic bone metastases	2.451 ± 0.032	Osteolytic bone metastases	2.470 ± 0.024
Healthy (Day 84)	2.420 ± 0.013	Healthy (Day 84)	2.472 ± 0.012
Osteoblastic bone metastases	2.473 ± 0.039	Osteoblastic bone metastases	2.497 ± 0.021

Second harmonic generation imaging on H&E stained histology slides derived susceptibility ratio values of untreated and treated femora of healthy and bone with metastases. Std.: standard deviation.

### Backscatter analysis

**Fig 5:**
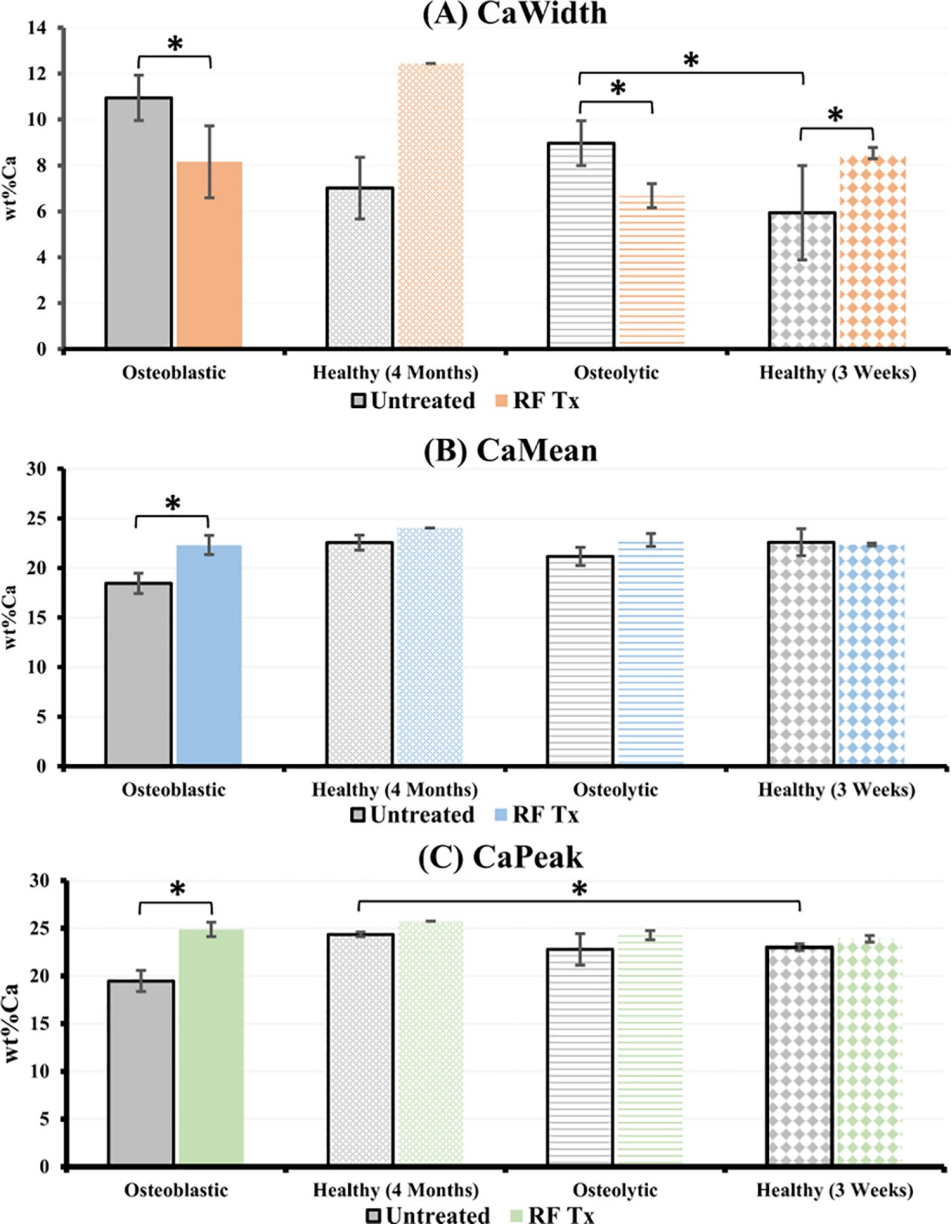
Mineralization profile of bone tissue. RFA-induced bone mineral density distribution (BMDD) changes in healthy femora and femora with metastatic lesions calculated from BSE images. The mineralization profile (a) Calcium Width (CaWidth) b) Calcium Mean (CaMean) and c) Calcium Peak (CaPeak)) of healthy and bone with metastatic lesions is modified post treatment. * Represents a significant difference between indicated groups (p < 0.05).

The mineral distribution profile of the rat femoral trabecular bone was impacted secondary to RFA treatment in metastatic and healthy cohorts. The reduced mineral content and increased heterogeneity of osteoblastic bone tissue was demonstrated previously in osteoblastic regions of a mixed metastatic model [31]. Untreated Bone with osteolytic lesions demonstrated higher heterogeneity compared to untreated age-matched healthy bone (Day 21: 8.968 vs.5.941, p = 0.024, Fig 5A). Homogeneity of untreated healthy bone tissue was not significantly different between day 84 and 21 (Day 84: 7.014 vs. Day 21: 5.941, p = 0.448, Fig 5A). Average mineral content did not exhibit significant differences between untreated healthy rats at day 84 and 21 (Day 84 vs. Day 21: 22.553 vs. 22.601, p = 0.957, Fig 5B). However, younger untreated healthy bone (Day 21) has significantly lower maximum peak intensity in comparison to untreated healthy bone at day 84 (Day 84: 24.365 vs. Day 21: 23.03, p = 0.009, Fig 5C).

Older healthy animals (Day 84) did not display significantly different levels of mineralization post treatment (CaPeak: 24.365 vs. 25.747, p = 0.057, Fig 5C; CaMean: 22.553 vs. 24.031, p = 0.299, Fig 5B). However, young healthy rats (Day 21) displayed an increased heterogeneity post-RFA (8.539 vs. 5.941, p = 0.001, Fig 5A). RFA-treated osteoblastic bone also demonstrated a significantly increased homogeneity and mineralization profile compared to the untreated osteoblastic bone tissue (CaWidth: 8.158 vs. 10.947, p = 0.001, Fig 5A; CaMean: 22.315 vs. 18.450, p = 0.001, Fig 5B; CaPeak: 24.889 vs. 19.479, p = 0.001, Fig 5C). In the osteolytic model a significant difference was only found in the heterogeneity of the bone post-RFA (6.680 vs. 8.968, p = 0.043, Fig 5A). Mineralization profiles of the RFA-treated osteoblastic and osteolytic bone did not demonstrate any significant differences compared to the corresponding untreated, age-matched, healthy animals (CaMean, Day 84: 22.315 vs. 24.031, p = 0.104; Day 21: 22.839 vs. 22.362, p = 0.764, Fig 5B; CaPeak, Day 84: 24.889 vs 25.747, p = 0.137; Day 21: 24.269 vs 23.891, p = 0.068, Fig 5C).

## Discussion

Repeatable thermal lesions were created via RFA at target RF temperature of 65 ˚C in healthy rat femora and femora with osteoblastic and osteolytic bone lesions. Thermal monitoring demonstrated physiologically normal temperatures on the skin surface and macroscopically no damage to the tissue surrounding the femur was observed. The impact of RFA on bone quality, in healthy bone tissue and secondary to osteolytic and osteoblastic metastases, was characterized using histological analyses and assessment of changes in mineral and organic phases of bone tissue.

Histological findings, post-RFA, provided a groundwork for numerous observations. Post inoculation, in metastatic bone tissue and corresponding age-matched healthy cohorts (Day 21 and Day 84), osteocytes (live and dead) were present in abundance in the ablation zone (Figs 3 and 4). Frank necrosis was observed in the bone marrow, however, repopulation of hematopoietic (bone marrow) cells was preserved [36]. Based on radiological and histological findings, the general morphology of femora did not indicate any RFA-induced structural damage. In addition, histological observations did not demonstrate any evidence of cortical thinning, congruent with the literature [37]. Histological findings also demonstrated formation of fibrous tissue which is a typical response to an RFA procedure. hEGFr immunohistology staining tumor cells, present in samples from osteolytic bone metastases, showed the drastic impact of coagulation necrosis in treatment of tumorous cells post-RFA (Fig 4).

Impact of RFA on bone quality was assessed in both the mineral and organic phases of bone. In the metastatic and age-matched healthy cohorts, trabecular collagen fibril organization analyzed using SHG was impacted in the presence of RFA. Proliferation of osteoblasts/osteoclasts was observed at the periphery of the ablated region coupled with the presence of highly disorganized woven bone. In comparison to untreated age-matched healthy rats (Day 21 and 84), SHG intensities of corresponding RFA-treated healthy bone tissue (Day 21 and 84) displayed an increased susceptibility ratio, indicating a reduced degree of collagen fibril organization (Table 1) [38]. There are several factors that may be modified due to the RFA dose delivery which results in the observed increase in disorganization of the collagen fibrillar network. This may be due to the pre-existing (before RFA treatment) higher degree of collagen fibril disorder associated with metastatic involvement [27, 29]. Interactions between collagen fibrils, fibrillogenesis and degree of fibril organization may be impacted [23, 39–41] by factors such as fibril-associated collagen with interrupted triple helices’ (FACITs) concentration, modified acidity and concentration of leucine-rich proteoglycans (SLRPs) [27, 30, 42–45] caused by RFA-induced modifications in bone tissue. With higher pre-treatment susceptibility ratio values in the metastatically involved groups, due to the already established presence of metastatically induced disorganization of collagen fibrils, there was no evidence of significant further collagen fibril disorganization post-RFA.

BMDD of healthy and metastatic bone is modified post-RFA. In healthy bone, regardless of the age of the model (Day 21 or Day 84), average mineral content is unchanged post-treatment. However, younger healthy bone (Day 21), demonstrates greater heterogeneity post treatment which may be due to the shorter recovery period. In the osteolytic bone metastases model, due to the aggressive nature of tumor growth, longer observation periods are not possible. In comparison to older healthy bone (Day 84), which is monitored for 28 days post-treatment and hence undergoes several remodeling cycles; age-matched younger healthy bone (Day 21) is only monitored for 7 days post-treatment (a complete bone remodeling cycle normally takes two about weeks in rats) [46]. Several studies have reported stimulated new bone formation, particularly at the ablation zone’s periphery post RFA [10, 12, 32, 47–49]. The RFA-induced newly formed bone in the healthy femora of younger rats (Day 21) would also not be as developed as the RFA-induced formed bone within the older healthy bone (Day 84). These findings support the lower maximum peak intensity observed in the younger healthy bone tissue (Day 21).

Post-RFA treatment, osteoblastic bone tissue demonstrated an enhanced mineralization profile with improved homogeneity. Higher mineralization values (CaPeak) and elevated mineral content (CaMean) are observed post-RFA (Fig 5). It is evident from the histopathological analyses that RFA interrupts metastatic cell activity locally. Impaired osteoblastic-induced formed bone is replaced with RFA-induced bone which is going under normal bone remodeling cycle. New hydroxyapatite (HA) deposits are reflected in reported enhanced calcium weight percentage. Trabeculae of the newly formed, RFA-induced bone are like the rest of the trabecular network in the femoral cavity in terms of architecture, connectivity and mineral content resulting in an overall more homogenous mineralization profile. In addition, the bone remodeling cycle and subsequent mineralization not only occurs in phases but is also time-dependent [50]. Like the age-matched healthy group, the osteoblastic model was monitored for 28 days post-RFA treatment. During this time, newly formed RFA-induced bone reaches later stages of bone mineralization resulting in appearance of more mature bone, which contributes to the improved mineralization profile. In bone with osteolytic lesions, the mineralization profile is also improved post-RFA delivery via enhanced homogeneity, however, increased average mineral content was not observed post-RFA treatment which may be due to the shorter recovery time associated with this model. Finally, the mineralization profile of the RFA-treated metastatic models did not show any significant differences compared to the corresponding RFA-treated, age-matched healthy animals (Day 21 and Day 84). Newly formed bone replaces the formerly impacted metastatic bone and undergoes remodeling cycle(s). This results in the formation of new bone which more closely mimics the mineralization of healthy bone. This suggests a positive impact of RFA delivery on bone quality in the metastatic femur.

Our earlier work evaluating bone quality secondary to skeletal metastatic involvement has considered Raman spectroscopy in addition to SHG and BSE to assess changes in bone quality in pathologic vertebrae [27]. In this work SHG and BSE were chosen as they provide quantitative rather than relative measures of changes (mineral/matrix ratio) in the organic and mineral phases of the bone. Additional endpoints for microdamage and load-to-failure analyses will be considered in future work.

## Conclusion

In this study, the impact of RFA on the mineral and organic components of healthy and metastatic bone were assessed. In the context of skeletal metastases, RFA resulted in a more homogenous bone tissue by replacing the diseased bone with more mature, highly mineralized bone. RFA treatment was shown to be effective in targeting tumor cells and promoting subsequent new bone formation. RFA did not negatively impact healthy bone tissue. The positive impact of RFA on bone quality in metastatically involved bone is important in directing the utilization of this growing local minimally invasive therapeutic approach with respect to fracture risk assessment, patient selection and multimodal treatment planning.
